# Interventions to promote technology adoption in firms: A systematic review

**DOI:** 10.1002/cl2.1181

**Published:** 2021-11-03

**Authors:** David Alfaro‐Serrano, Tanay Balantrapu, Ritam Chaurey, Ana Goicoechea, Eric Verhoogen

**Affiliations:** ^1^ Cornerstone Research New York New York USA; ^2^ World Bank Group Washington District of Columbia USA; ^3^ School of Advanced International Studies (SAIS) Johns Hopkins University Washington District of Columbia USA; ^4^ Department of Economics and School of International and Public Affairs Columbia University New York New York USA

## Abstract

**Background:**

The adoption of improved technologies is generally associated with better economic performance and development. Despite its desirable effects, the process of technology adoption can be quite slow and market failures and other frictions may impede adoption. Interventions in market processes may be necessary to promote the adoption of beneficial technologies. This review systematically identifies and summarizes the evidence on the effects of interventions that shape the incentives of firms to adopt new technologies. Following Foster and Rosenzweig, *technology* is defined as “the relationship between inputs and outputs,” and *technology adoption* as “the use of new mappings between input and outputs and the corresponding allocations of inputs that exploit the new mappings.” The review focuses on studies that include direct evidence on technology adoption, broadly defined, as an outcome. The term intervention refers broadly to sources of exogenous variation that shape firms' incentives to adopt new technologies, including public policies, interventions carried out by private institutions (such as NGOs), experimental manipulations implemented by academic researchers trying to understand technology adoption, and natural experiments.

**Objective:**

The objective of this review is to answer the following research questions:

1. To what extent do interventions affect technology adoption in firms?
2. To what extent does technology adoption affect profits, employment, productivity, and yields?
3. Are these effects heterogeneous across sectors, firm size, countries, workers' skill level, or workers' gender?

**Selection Criteria:**

To be included, papers had to meet the inclusion criteria described in detail in Section 3.1 which is grouped into four categories: (1) Participants, (2) Interventions, (3) Methodology, and (4) Outcomes.

Regarding participants, our focus was on firms, and we omitted studies at the country or region level. In terms of interventions, we included studies that analyzed a source of exogenous variation in incentives for firms to adopt new technologies and estimated their effects. Thus, we left out studies that only looked at correlates of technology adoption, without a credible strategy to establish causality, and only included studies that used experimental or quasi‐experimental methods. Regarding outcomes, papers were included only if they estimated effects of interventions (broadly defined) on technology adoption, although we also considered other firm outcomes as secondary outcomes in studies that reported them.

**Search Methods:**

The first step in selecting the studies to be included in the systematic review was to identify a set of candidate papers. This set included both published and unpublished studies. To look for candidate papers, we implemented an electronic search and, in a subsequent step, a manual search.

The electronic search involved running a keyword search on the most commonly used databases for published and unpublished academic studies in the broad topic area. The words and their Boolean combinations were carefully chosen (more details in Section 3.2). The selected papers were initially screened on title and abstract. If papers passed this screen, they were screened on full text. Those studies that met the stated criteria were then selected for analysis.

The manual search component involved asking for references from experts and searching references cited by papers selected through the electronic search. These additional papers were screened based on title and abstract and the remaining were screened on full text. If they met the criteria they were added to the list of selected studies.

**Data Collection and Analysis:**

For the selected studies, the relevant estimates of effects and their associated standard errors (*SE*s) were entered into an Excel spreadsheet along with other related information such as sample size, variable type, and duration for flow variables. Other information such as authors, year of publication, and country and/or region where the study was implemented was also included in the spreadsheet.

Once the data were entered for each of the selected studies, the information on sample size, effect size and *SE* of the effect size was used to compute the standardized effect size for each study to make the results comparable across studies. For those studies for which relevant data were not reported, we contacted the authors by email and incorporated the information they provided. Forest plots were then generated and within‐study pooled average treatment effects were computed by outcome variable.

In addition, an assessment of reporting on potential biases was conducted including (1) reporting on key aspects of selection bias and confounding, (2) reporting on spillovers of interventions to comparison groups, (3) reporting of *SE*s, and (4) reporting on Hawthorne effects and the collection of retrospective data.

**Results:**

The electronic and manual searches resulted in 42,462 candidate papers. Of these, 80 studies were ultimately selected for the review after screenings to apply the selection criteria. Relevant data were extracted for analysis from these 80 studies. Overall, 1108 regression coefficients across various interventions and outcomes were included in the analysis, representing a total of 4,762,755 firms. Even though the search methods included both high‐income and developing countries, only 1 of the 80 studies included in the analysis was in a high‐income country, while the remaining 79 were in developing countries.

We discuss the results in two parts, looking at firms in manufacturing and services separately from firms (i.e., farms) in agriculture. In each case, we consider both technology adoption and other firm outcomes.

**Authors' Conclusions:**

Overall, our results suggest that some interventions led to positive impacts on technology adoption among firms across manufacturing, services, and agriculture sectors, but given the wide variation in the time periods, contexts, and study methodologies, the results are hard to generalize. The effects of these interventions on other firm performance measures such as farm yields, firm profits, productivity, and employment were mixed.

Policy‐makers must be careful in interpreting these results as a given intervention may not work equally well across contexts and may need to be adjusted to each specific regional context. There is great need for more research on the barriers to technology adoption by firms in developing countries and interventions that may help alleviate these obstacles. One major implication for researchers from our review is that there is a need to carefully measure technology adoption.

## PLAIN LANGUAGE SUMMARY

**Interventions to promote technology adoption in firms: limited evidence of positive effects**

Some interventions lead to an increase in technology adoption among firms across manufacturing, services, and agriculture sectors, but these effects are context‐specific, as well as intervention‐specific. The effects of these interventions on other firm performance measures such as farm yields, firm profits, productivity, and employment are mixed.

**What is this review about?**

This review summarizes the evidence on the effects of interventions that may affect technology adoption, such as providing firms with training or grants, or a change in a trade policy. Interventions can be carried out by governments, private institutions (such as NGOs), or researchers trying to understand technology adoption, or they can occur as natural experiments.

**What is the aim of this review?**

This Campbell systematic review seeks to answer three questions: To what extent do interventions affect technology adoption in firms? To what extent does technology adoption affect profits, employment, productivity, and yields? Are these effects heterogeneous across sectors, firm size, countries, workers' skill level, or workers' gender?

**What studies are included?**

Included studies had to analyze firms, and examine the effects of an intervention. Studies at country or regional level were omitted.

Interventions were broadly defined, including the direct provision of funding for technological adoption (Direct Financial Support), support to pay for the cost of the adoption projects without directly providing funding (Indirect Financial Support), nonpecuniary interventions (Other Direct Support), and rules, policies, and characteristics of the environment that affect agents' incentives (Regulations and Standards).

Studies had to assess the causal effects of interventions with experimental and quasi‐experimental methods, excluding those that look at correlations. And they had to have technology adoption as the primary outcome of interest. This therefore excluded studies that do not present a measure of technology adoption.

Overall, 80 studies were included in the review, 79 analyzing effects of technology adoption in developing countries and one in a high‐income country. These studies analyzed the effects on technology adoption in 4,762,755 firms.

**What are the main findings of this review?**

Some interventions lead to positive impacts on technology adoption among firms, but these effects are context‐specific and intervention‐specific. In manufacturing and services, 19 of the 33 studies analyzed find positive and statistically significant standardized effects on technology adoption. In agriculture, 20 of the 47 studies analyzed find positive and statistically significant standardized effect sizes.

Most studies focused on analyzing the effects of “Other Direct Support,” which includes nonpecuniary interventions such as extension services, training, consulting, and SMS reminders. Overall, one group of interventions cannot be said to lead to a higher impact than others. Furthermore, the effects of these interventions on other firm performance measures such as farm yields, firm profits, productivity, and employment were mixed.

Due to the wide range of interventions and outcomes used across the studies analyzed, it is not possible to assess whether effects are similar across groups, or to calculate an average treatment effect across studies.

**What do the findings of this review mean?**

A statistically insignificant finding for a type of intervention in a particular context does not mean that all interventions of that type are not worthy of consideration. Policymakers should pay attention to how programmes can be improved and better tailored to particular environments, to achieve better outcomes.

Areas of future research could include both an understanding of barriers to technology adoption, and interventions that lead to increased adoption through removal of those barriers. This should analyze interventions that are less studied. For example, “Indirect Financial Support” including interventions such as access to credit, and incentive payments, as well as “Regulations and Standards” have been less studied for their effects on technology adoption.

Studies should also provide all the information required to compute standardized mean differences. They should also improve reporting on heterogeneous effects and the Hawthorne effect (in which the monitoring process itself affects behavior).

**How up‐to‐date is this review?**

The review authors searched for studies between 2000 and 2020.

## 1 BACKGROUND

### 1.1 The policy issue

The adoption of improved technologies is generally associated with better economic performance and development. Governments and development agencies have incorporated the promotion of firms' competitiveness into their priorities (IADB, 2016; World Bank, 2017) and recognized that the adoption of modern technologies is one of its drivers. Two reasons lie behind this interest in competitiveness in general and technological upgrading in particular: first, the expectation that technological upgrading by firms will deliver benefits like higher productivity, more jobs, better wages and better working conditions at the micro level, and higher growth at the aggregate level; and second, the idea that the slowness, or even absence, of the process of technology adoption is due in part to market failures due to externalities, imperfect information, and coordination problems that call for public intervention. Furthermore, these market failures are often more salient in developing countries, leading to lower rates of new technology adoption, and consequently slower growth rates.

History provides examples of major increases in wellbeing broadly defined (including output, output per worker, size, and productivity) associated with the widespread adoption of improved productive methods. Cirera et al. (2017) point to the so‐called three industrial revolutions in manufacturing: the introduction of steam‐powered machines, the adoption of electricity‐powered production methods, and the use of information technologies to automate manufacturing. In agriculture, the green revolution, which involved the adoption of new technologies, including high‐yielding varieties, and the increased use of fertilizers and pesticides, had a comparable impact. The key aspect in these revolutionary transformations was not the mere invention of new technologies, but their widespread adoption by firms.

Despite its desirable effects, the process of technology adoption can be quite slow (Geroski, 2000; Rosenberg, 1972) and market failures and other frictions may impede adoption (Foster & Rosenzweig, 2010; Verhoogen, 2020). Such market failures and frictions impeding technology adoption may also be different for agricultural firms compared to nonagricultural firms. For example, information frictions may be more significant for nonagricultural firms than for agricultural firms. This may in part be because agricultural firms can observe technology adoption by neighboring firms, but this is often not possible for nonagricultural firms. Furthermore, nonagricultural firms seldom share information on new beneficial technology with competing firms. Thus, interventions in market processes may be necessary to promote the adoption of beneficial technologies.

The lack of technology adoption in cases in which the potential gains are clear have been observed in specific industries like textiles (Bloom et al., 2013) and soccer‐ball production (Atkin et al., 2017). Foster et al. (2008) show that there is large heterogeneity in productivity even within narrowly defined industries, which may be the consequence of the lack of technology adoption by the firms in the left tail of the distribution.

To help inform governments' actions, this systematic review aggregates the existing evidence on interventions that induce technology adoption and their implications for firm outcomes.

### 1.2 Potential channels of effects

We think of the effects of interventions as occurring in two stages. In the first stage, interventions may induce the adoption of better technologies in the directly affected firms, provided that that the technologies are indeed better (after considering adoption costs) and that, after providing the treatment, firms are able to notice that advantage. There are three broad categories of reasons for low technology adoption (i) internal to the firm, (ii) on the input side, and (iii) on the output side (Verhoogen, 2020). First, there may be low adoption of new technologies because firms may not be profit maximizing, may lack information, or may not have the capability to apply the information in practice. These are factors internal to the firm. Second, on the input side, newer technology often requires highly skilled workers and high‐quality inputs, which may be scarce and expensive. Finally, on the output side, demand conditions may affect the incentives of firms to adopt (or not) new technologies. For example, richer customers may have preferences for higher‐quality goods, which may require the application of new technologies.

In a second stage, the adoption of a better technology by treated firms may lead to an increase in total output, output per unit of input, unit cost, firm‐level wages, employment, total factor productivity, firm survival, and/or exports. Productivity, understood as the inverse of cost, is expected to increase because the reduction in (quality‐adjusted) cost is presumably what makes the new technology attractive to the firm. Output is expected to increase if a firm can increase its market share based on the reduction in costs. As consequence of the expansion in output, firm‐level employment is also generally expected to increase. Wages (although not necessarily wages relative to other input prices) may also go up after adoption due to the rise in marginal labor productivity. In general, we expect a new technology to lead to an increase in inputs in production. However, we note that it is theoretically possible that some technological innovations may be input‐saving; that is, they may be labor‐, capital‐, or land‐saving. In fact, because of the possibility that a new technology may be input‐substituting, studies often report multiple firm performance indicators such as profits, and productivity along with changes in inputs (labor, capital, land).

**Figure 1:**
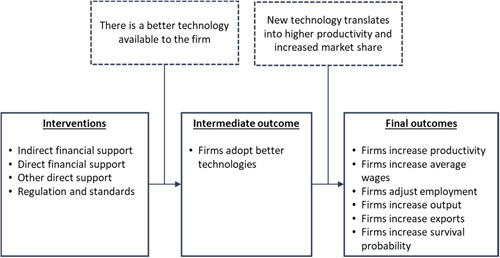
Potential channels of effects

Figure 1 presents the theory of change of the interventions of interest. The solid boxes represent the intervention and outcomes and the dashed boxes represent the assumptions. As stated before, this review is not devoted to a single intervention or technology‐adoption outcome. Instead, it includes several interventions inducing technology adoption in firms (measured in several ways). Despite these differences, it is useful to provide a general theory of change describing the way in which the interventions, intermediate outcomes, and final outcomes relate.

### 1.3 Why it is important to do the review

There is a need to aggregate evidence across multiple studies concerning similar economic phenomenon to translate research into policy (Allcott, 2015; Banerjee et al., 2015; Dehejia, 2015; Meager, 2019). A systematic review allows one to survey the literature on a given economic phenomenon, identify the knowledge gaps, and compare effects across contexts, implementers and time, facilitating the integration of research into policy.

Systematic reviews about technology adoption are mostly focused on agricultural firms (Fuglie et al., 2019; Obayelu et al., 2017; Silva et al., 2015; Waddington et al., 2014). There has been little effort to do the same for other economic sectors, despite the recent publication of high‐profile papers on technology adoption in manufacturing (e.g., Atkin et al., 2017; Bloom et al., 2013). Consequently, existing summaries of evidence for nonagricultural firms are almost all nonsystematic literature reviews. Our review addresses this knowledge gap by summarizing the existing evidence on technology adoption in nonagricultural as well as agricultural firms.

A review close to ours is Piza et al. (2016). The authors systematically review the evidence about the impact of businesses‐support services for small and medium enterprises (SMEs) in low and middle‐income countries. Our review complements their findings by focusing specifically on technology‐adoption outcomes for a broader set of interventions and a broader set of firms and countries. Including a broader set of firms is important because, even though SMEs are particularly relevant for outcomes like employment, larger firms are key for other important outcomes like exports. Considering also the experience of high‐income^1^ countries is warranted because active policies to promote technology adoption are widespread around the globe, not only in the developing world. The experience of these countries is relevant for policy making everywhere. There are also aspects of the technology‐adoption process, for instance knowledge spillovers across firms (Hausmann & Rodrik, 2003), that carry distinctive implications for the design of policy but that arguably are not as salient in the context of other pro‐business interventions, such as programs to increase formalization and access to working capital.

Existing reviews on technology adoption in nonagricultural firms that do not follow the Campbell guidelines for systematic reviews include Herbert‐Copley (1990), Keller (2004), Oliveira and Martins (2011), and Foster and Rosenzweig (2010). Herbert‐Copley (1990) reviews case studies of technical change in manufacturing firms in the 1980s in Latin America. The study assesses the role of the nature of technology, market structure, government policy, firm characteristics and the location of the international technological frontier on the level of technology adoption. Keller (2004) focuses at an aggregate level and on diffusion across countries. Coming from the information systems literature, Oliveira and Martins (2011) compile studies on the adoption of information technology at firm level. Their review does not attempt to compare the results across studies. Close in spirit to the current review, Foster and Rosenzweig (2010) review microeconomic studies of the barriers to technology adoption in low‐income countries. Among the factors they find to be important are financial and nonfinancial returns to adoption, individual learning and social learning, technological externalities, scale economies, schooling, credit constraints, risk and incomplete insurance, and departures from behavioral rules implied by simple models of rationality. The current review differs in the systematic search to select studies of interest, and in its coverage of more recent work.

## 2 OBJECTIVE

The primary objective of this review is to assess the extent to which interventions affect technology adoption in firms. The secondary objectives are to assess to what extent technology adoption affects other firm outcomes and whether these effects differ across certain groups.

In particular, the review aims to answer the following research questions:

1. To what extent do interventions affect technology adoption in firms?
2. To what extent does technology adoption affect profits, employment, productivity, and yields?
3. Are these effects heterogeneous across sectors, firm size, countries, workers' skill level, or workers' gender?

Question 1 refers to the immediate impact of interventions to promote technology adoption. Question 2 explores the subsequent impact of technology adoption on other economic outcomes. Question 3 explores heterogeneous effects across relevant groups.

## 3 METHODS

### 3.1 Criteria for considering studies for this review

After conducting predefined manual and electronic searches which used keywords and imposed restrictions in terms of years and language covered, we applied screening criteria to identify studies to be included in the review. These criteria were grouped into four categories described in detail below and in the published review protocol (Verhoogen et al., 2018).

#### 3.1.1 Types of participants considered

To be included in this review, studies must have firms as the unit of analysis. The term “firm” refers to productive units of any size, including single‐person businesses and farmers or farms. This focus leaves out studies of technology adoption at the country or region level.

This review does not impose any restriction regarding the level of development of the country in which the intervention takes place. It includes studies in both high‐income and developing countries.

#### 3.1.2 Types of interventions considered

This review focuses on interventions that induce adoption of a new technology by a firm. Following Foster and Rosenzweig (2010), we define *technology* as “the relationship between inputs and outputs,” and *technology adoption* as “the use of new mappings between input and outputs and the corresponding allocations of inputs that exploit the new mappings.” This definition of technology adoption is broad, and includes the overall production plan that firms implement as well as changes in specific practices.

The term intervention refers broadly to sources of exogenous variation that shape firms' incentives to adopt new technologies, including public interventions, interventions carried out by private institutions (such as NGOs), experimental manipulations deliberately induced by academic researchers trying to understand technology adoption, and natural experiments. For instance, Bloom et al. (2016) study how the inclusion of China into a trade agreement allowing Chinese imports into Europe impacts technology adoption among firms. In this case, we consider the inclusion in the trade agreement as an intervention.

Although this review is not focused on a particular type of policy, it is possible to provide a broad classification of the interventions we expect to find. Here we follow the classification presented by Cirera and Maloney (2017):

- **Direct financial support.** These are interventions that directly provide funding for technological adoption. This assistance could take many forms, including access to credit, insurance, subsidies, in‐kind transfers, or cash towards the take‐up of technology. There are studies that explore the impact of access to credit to firms (Giné & Dean, 2009), access to insurance (Karlan et al., 2014), in‐kind provision of equipment (Atkin et al., 2017; de Mel et al., 2012), and/or cash (Chudnovsky et al., 2011).
- **Indirect financial support.** These are interventions that help businesses to pay for the cost of the adoption projects without directly providing the resources. Loan guarantees (Tan, 2009), minimum support price (Abate et al., 2018), and changing policy on the supply chain to reduce the price of inputs (Ogunniyi et al., 2017) are examples of interventions in this category.
- **Other direct support.** These are nonpecuniary interventions, including policies implemented by governments such as technology extension services, and experimental interventions such as the direct provision of management consultancy services (Bloom et al., 2013; Bruhn et al., 2018), awareness interventions (Beaman et al., 2014), provision of information (Ali & Rahut, 2013; Beaman et al., 2018; Benyishay et al. (2016); Kondylis et al., 2017), and training (Feder et al., 2003; Field et al., 2010; Karlan & Valdivia, 2011).
- **Regulation and standards.** These are rules, policies, and characteristics of the environment that affect agents' incentives, for example a trade policy that induces technology adoption (Bloom et al., 2016), or a positive demand shock to technology adoption induced by a government regulation (Crouzet et al., 2019; Higgins, 2019).

While the categories above each represent a broad set of interventions, studies may combine interventions across the categories. For instance, a government policy that helps subsidize take‐up of a new input and provides training can be classified under both “direct financial support” and “indirect financial support” (e.g., Ogunniyi et al., 2017). Similarly, a study that includes provision of loans, as well as training to microfinance clients (e.g., Gine & Mansuri, 2014) can be classified under “direct financial support” and “other direct support.”

It is important to note that the focus of this review is the effect of the interventions on technology adoption by *existing* firms. In this sense, the interventions considered are different from interventions to promote entrepreneurship. We expect that most of the studies are about the adoption of technologies that are new to the firm, but not to the world.

Papers that do not focus on an intervention (defined broadly as above) are not included in this review. For example, the seminal papers by Conley and Udry (2010) and Tavneet Suri (2011), while important and influential for the broader literature, do not focus on an explicit intervention (defined as above) and have therefore been excluded.

#### 3.1.3 Methodologies considered

The review includes studies that take the firm as the unit of analysis and employ an identification strategy explicitly aiming to estimate causal effects on technology adoption. Experimental and quasi‐experimental methods of causal inference attempt to disentangle the effect of the intervention of interest on a given outcome from the effect of omitted variables that also affect the outcome and correlate with the intervention. If such omitted variables are present, their influence on the outcome will be confounded with the influence of the intervention and the analyst could end up attributing to the treatment what is really the result of the omitted variable.

Randomized control trials (RCTs) are commonly perceived to be the gold standard in conducting causal inference. But RCTs are costly to run and often infeasible. In addition to experimental studies, we include papers using quasi‐experimental methods, including instrumental variables, regression‐discontinuity designs, difference‐in‐differences, and propensity‐score matching.

We generally require that the comparison group is a set of firms that received no treatment. Two sorts of exceptions to this rule are considered acceptable. One is when different groups receive exogenously different “doses,” or amounts, of treatment, but no group receives zero treatment. The other is when the researchers provide compensation to the comparison group to replicate the nonintervention situation or to isolate some aspect of the treatment. In these cases, it is still possible to estimate causal effects cleanly, and hence they are considered in the review.

#### 3.1.4 Types of outcome measures considered

The primary outcome of interest for the review is technology adoption. Measures of adoption can take the form of dichotomous variables (that, e.g., takes value one when a technology is adopted and zero otherwise) or a count of the number of new practices adopted. Because the primary objective of this review is to examine the effects of interventions on technology adoption, we exclude papers that do not consider a technology adoption outcome.

The secondary outcomes are other firm outcomes that may be affected by technology adoption. These are variables in the second step of the causal chain. In particular, we consider measures of output per unit of input, unit cost, wages, employment, output, total factor productivity, and profits. Regarding measurement, we require that (i) output and output per worker are measured in physical or monetary units; (ii) wages are measured in monetary units; and (iii) employment is measured in hours or days or number of employees. Total factor productivity is treated as a dimensionless variable.

Papers analyzing effects on secondary outcomes are included only if they also present estimates for the primary outcome (technology adoption).

### 3.2 Search methods for identification of studies

The first step in selecting studies for the review was to identify a set of candidate papers. This set includes both published and unpublished studies. To look for candidate papers, we implemented an electronic search and, in a subsequent step, a manual search.

#### 3.2.1 Electronic search

The electronic search included bibliographic databases and specialized journals. We also looked for studies on the websites of key organizations to ensure good coverage of unpublished literature.

*Bibliographic databases*:

1. Academic Search Complete (through EBSCO Host).
2. Business Source Complete (through EBSCO Host).
3. EconLit (through EBSCO Host).
4. Ideas Repec (through EBSCO Discovery).
5. PAIS Index (through ProQuest).
6. ProQuest Dissertations & Theses Global.

*Websites of key organizations*:

1. 3ie database of impact evaluations.
2. African Development Bank.
3. Agricultural Technology Adoption Initiative (ATAI).
4. American Economic Association RCT Registry.
5. Asian Development Bank.
6. Inter‐American Development Bank.
7. Organizations for the Economic Co‐operation and Development (OECD).
8. UK Department for International Development (DFID).
9. US Agency for International Development (USAID).
10. World Bank Group.

Our electronic search used Boolean operators to express the following requirements:

1. The studies should include terms related to the outcomes of interest for the study in the title or abstract.
2. The studies should include terms related to the methodology employed in the title, the abstract, or the main text.
3. The papers should be dated in the year 2000 or later. Similar to Piza et al. (2016), we impose this limit because we focus on studies using impact‐evaluation techniques, which have been widely adopted in development economics since that time. Even though it is possible that some papers on technology adoption were produced before this year, we expect that few address endogeneity issues in the way required to be included in this review.

To keep the electronic search as broad as possible, we did not differentiate between our primary outcomes (technology adoption) or secondary outcomes (other firm outcomes). The keyword searches were piloted multiple times on at least two different databases to ensure the keywords were able to identify the right set of papers. The pilots were assessed based on whether some of the manually selected seminal papers would show up when conducting keyword searches. The final list of key words was selected once we had found the combination to be working on at least two different databases.

In each electronic database, we first imposed filters for publication date on or after 2000 and English language. The language filter was mostly due to resource constraints and the year filter to the fact that methodologies analyzing causal effects of exogenous variations were not popular before 2000. Then, following EBSCO host's search syntax, the following Boolean expression was implemented:

(TI(*outcomes*) OR AB(*outcomes*)) AND (TI(methods) OR AB(methods) OR TX(methods))

The exact syntax used in EBSCO host, ProQuest, and Ideas Repec through EBSCO Discovery is:

(TI(((technolog* OR manag* OR innovat* OR practice*) W4 (adopt* OR diffus* OR chang* OR alter*)) OR productivity OR “output per” OR (unit* W4 cost)) OR AB(((technolog* OR manag* OR innovat* OR practice*) W4 (adopt* OR diffus* OR chang* OR alter*)) OR productivity OR “output per” OR (unit* W4 cost))) AND (TI((“quasi experiment*” or quasi‐experiment* or “random* control* trial*” or “random* trial*” or RCT or (random* N3 allocat*) or match* or “propensity score” or PSM or “regression discontinuity” or “discontinuous design” or RDD or “difference in difference*” or difference‐in‐difference* or “diff in diff” or “case control” or cohort or “propensity weighted” or propensity‐weighted or “interrupted time series” or “Control* evaluation” or “Control treatment” or “instrumental variable*” or heckman or IV or ((quantitative or “comparison group*” or counterfactual or “counter factual” or counter‐factual or experiment*) N3 (design or study or analysis)))) OR AB((“quasi experiment*” or quasi‐experiment* or “random* control* trial*” or “random* trial*” or RCT or (random* N3 allocat*) or match* or “propensity score” or PSM or “regression discontinuity” or “discontinuous design” or RDD or “difference in difference*” or difference‐in‐difference* or “diff in diff” or “case control” or cohort or “propensity weighted” or propensity‐weighted or “interrupted time series” or “Control* evaluation” or “Control treatment” or “instrumental variable*” or heckman or IV or ((quantitative or “comparison group*” or counterfactual or “counter factual” or counter‐factual or experiment*) N3 (design or study or analysis))))OR TX((“quasi experiment*” or quasi‐experiment* or “random* control* trial*” or “random* trial*” or RCT or (random* N3 allocat*) or match* or “propensity score” or PSM or “regression discontinuity” or “discontinuous design” or RDD or “difference in difference*” or difference‐in‐difference* or “diff in diff” or “case control” or cohort or “propensity weighted” or propensity‐weighted or “interrupted time series” or “Control* evaluation” or “Control treatment” or “instrumental variable*” or heckman or IV or ((quantitative or “comparison group*” or counterfactual or “counter factual” or counter‐factual or experiment*) N3 (design or study or analysis)))))

“W4” is a search syntax that stands for “within 4 words of each other.” Similarly, “N3” stands for “near 3 words of each other.”

For other journal websites the syntax was adapted as appropriate. The electronic searches were run on May 2nd and 3rd, 2018.

#### 3.2.2 Manual search

In addition to the electronic search, we conducted a manual search implemented between May, 2018 and June, 2020. It entailed the following three procedures, also explained in detail in Appendix A:

- Recommendations: We sought recommendations of additional references from experts and practitioners.
- Backward‐citation search: for papers that met the criteria and were selected initially, we conducted a backward‐citation search in Google Scholar and all papers that cited the initially selected papers were screened.
- Reference search: for the initially selected papers, we screened the papers that they cited.

### 3.3 Data collection and analysis

#### 3.3.1 Screening procedure

**Figure 2:**
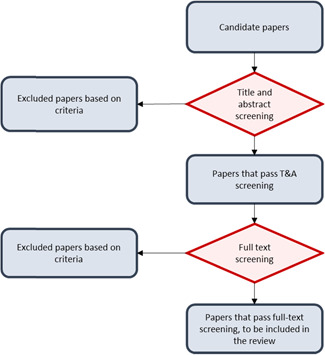
Screening procedure

The output of the electronic and manual searches was a very broad set of candidate papers (42,462), including many articles that were not relevant for our review. We used a two‐step screening procedure to identify papers that met the criteria for the review (see Figure 2):

*Step 1—Title and abstract screening*:

All candidate papers were screened based on their title and abstract. Candidate papers were grouped in batches of 350. Initially, two batches were double‐screened, with two reviewers screening each paper. Each reviewer applied the criteria independently and once they completed a batch of 350, their assessments were compared and discussed before they continued to the second batch. In the discussion, areas of disagreement were resolved.

The objective of the double‐screening procedure was to promote learning and increase consistency across reviewers. To move from double to single screening, we required a reduction in discrepancies between two reviewers in the second batch when compared to the first and in <5% of screened papers. After two batches (700 papers) were double‐screened in this way, this rule was met by all reviewers and each reviewer continued the title and abstract screening with single‐screening. Therefore, the remaining papers were screened only once.

*Step 2—Full text screening*:

Papers that passed the title and abstract screening were screened again based on the full article text. This time, to ensure consistency and learning across reviewers, we implemented the double‐coding procedure for the first 20 papers coded by each reviewer, grouped in two batches of 10.

In each of the screening stages, the reviewer had to determine whether a paper should be excluded or not based on the four elements of the criteria developed in Section 3.1, as follows:

1. Exclude based on participants. The study was excluded if the unit of analysis was not firms.
2. Exclude based on intervention. The study was excluded if it did not mention explicitly that the intervention had the potential of inducing technology adoption by a firm.
3. Exclude based on methodology. The study was excluded if it did not use one of the following methodologies: RCT, instrumental variables, regression discontinuity, difference‐in‐differences, propensity score matching or synthetic control.
4. Exclude based on outcomes. The study was excluded if it did not report the effect of the intervention on technology adoption. At this point, we did not impose restrictions regarding the secondary outcomes.

Papers that passed both screenings were selected for the data extraction phase. The two‐step screening procedure did not exclude articles based on whether the reported estimates were usable in the review or not.

Papers found manually in relevant websites as well as papers recommended by experts also went through the two‐step screening procedure.

Once a paper was selected for data extraction, the backward‐citation and reference searches described in Section 3.2 were conducted.

#### 3.3.2 Data extraction and management

Using the variables for data extraction presented in Appendix B, relevant information was entered in an Excel file for papers that passed the screening. This file included a coding sheet with basic characteristics at the study level such as citation, authors, year of publication, country, and methodology, among others. In addition, the file included a data analysis sheet with information at the regression level, including impact estimates, number of observations, and *SE*s.

A double‐entry procedure was carried out for a subset of the data from the selected papers (such as the impact estimates) to ensure the quality of the data entry. This was done for a randomly selected sample of 33% of the papers.

#### 3.3.3 Measures of treatment effect

Comparing different pieces of evidence about an intervention requires comparable effect‐size estimates. These comparable measures are usually not directly provided in the original study and need to be computed by the reviewer based on the information in the paper.

Different studies often measure outcomes in different units. For example, an outcome measure such as employment, may be expressed as number of workers, number of worker hours, number of hours per day, and so forth. This creates problems of comparisons across and within studies. Moreover, studies often use multiple indicators to express changes in relevant outcomes, exacerbating problems of comparison. As indicated in Borenstein et al. (2009), when the outcomes are not directly measured in a common and meaningful scale, one could use the standardized mean difference (SMD). This measure re‐expresses the impact measure of a study relative to the outcome variability observed in that study. A positive value of the SMD indicates a positive impact of the intervention on the outcome.

In several studies in our setting we were not able to find the breakdown of the sample between treatment and control groups. For this reason, as well as to ensure standardization across studies, we assume Nt = Nc = *N*/2. We then use the formula for SMD as provided in Waddington et al. (2019, p. 26):

$$\text{SMD}=\frac{2\left(\frac{\hat{\beta }}{SE},)\right)}{\sqrt{N}},$$

$$SE(\mathrm{SMD})=\sqrt{\frac{4}{N}+({\mathrm{SMD}}^{2}/4N)},$$

where $\hat{\beta }$ is the treatment effect, *SE* is the standard error and *N* is the sample size of the study.

#### 3.3.4 Unit of analysis issues

As discussed earlier, we focus on papers that take the firm as the unit of analysis. However, the unit of analysis might not coincide with the level of experimental or quasi‐experimental variation. For instance, a study might report estimates of firm‐level impacts of a technology adoption program that was assigned to the firms in selected districts. In this case, the treatment assignment status varies at the district level instead of the firm level. Studies like this should consider the intra‐cluster correlation when computing the *SE*s of their effect estimates. Fortunately, this practice has been increasingly adopted in social sciences with the use of cluster‐robust *SE*s. We did not have any studies that did not consider the intra‐cluster correlation while computing the *SE*s.

#### 3.3.5 Dealing with missing data

When relevant information is not reported (like the pooled standard deviations or sample sizes), we contacted the authors of the studies to ask for it. However, in nine cases we did not hear back or were unable to contact the authors. In such cases, we did not include the study in the analysis as we were unable to compute the SMD or the *SE*.

#### 3.3.6 Assessing reporting biases

We assessed reporting biases with a modified version of the 3ie risk of bias tool (Hombrados & Waddington, 2012), which is presented in detail in Appendix C. The tool covers four dimensions: (1) Reporting on key aspects of selection bias and confounding, (2) Reporting on spillovers of intervention to comparison groups, (3) Reporting of *SE*s, and (4) Reporting on Hawthorne effect and baseline data. If a study uses various methodologies, the assessments under selection bias and confounding are based on the most rigorous methodology.

For each of these dimensions, a set of considerations were defined. The question whether the study addresses each consideration can receive one of three answers: “reported,” “not reported,” or “not applicable.”

#### 3.3.7 Data synthesis

For the studies under consideration, we extracted available regression coefficients. In the context of multivariate regressions we extracted only the regression coefficients relevant to the outcomes we consider. All the effect sizes from the selected papers were converted into a standardized effect using the formula discussed above in the section on “Measures of treatment effect.” To avoid treating different results from the same paper as independent results, we computed an average standardized effect for all results, as well as the average *SE*s for these standardized effects within a paper for the same variable (Waddington et al., 2014).

The standardized effects and their respective *SE*s were computed manually. We generated forest plots to present the manually computed standardized effect sizes using the Stata command *metan*. The effect size as well as the confidence intervals for these outcomes are presented using a forest plot. It must be noted that we do not attempt to provide a summary effect across studies, because selected studies included several interventions and several definitions of technology adoption outcomes. Hence, we are not concerned with the computation of the summary effect, or the *SE* of the summary effect, commonly applied in other meta‐analyses/meta‐regressions.

#### 3.3.8 Deviation from protocol

The review has been executed to follow the steps described in the protocol (Verhoogen et al., 2018), with the following deviations:

- The protocol did not describe some of the manual searches conducted (reference search and citation search). These are now described in detail in Appendix A.
- The protocol included the following research questions to be answered by the review:
  1. To what extent do particular interventions affect technology adoption in firms?
  2. Is this effect heterogeneous across sectors, firm size, countries or owner's gender?
  3. To what extent does technology adoption affect total output, output per unit of input, unit cost, firm‐level wages, employment, total‐factor productivity, exports, and survival?
  4. Are these effects heterogeneous across sectors, firm size, countries, workers' skill level, or workers' gender?
  Few of the studies analyzed explore the effects on total output, output per unit of input, unit cost, firm‐level wages, exports, and survival as listed in Question 3 in the protocol. Therefore, it was not possible to analyze effects on these secondary outcomes. The research question was revised in this version to reflect the secondary outcomes analyzed (profits, employment, productivity, and yields).
- A double‐entry for a set of papers was conducted during data extraction as explained in Section 3.3, under “Data extraction and management.”
- We modified the tool for assessing risk of biases. We piloted the procedure described in the protocol and found that many studies received “unclear” classifications in the assessments. To avoid generating assessments that we felt might be misleading, we modified the tool to make it less subjective. In addition, we introduced the classifications “Not applicable” and “Not reported” instead of “Unclear.” The updated tool is reported in Appendix C and the assessments in Appendix D.
- Given that several measures of technology and several types of interventions were used in the selected papers, we decided that averages of effect sizes across studies would not be meaningful and we do not present them. We do present average effect sizes within studies as appropriate.

## 4 RESULTS

### 4.1 Description of studies

#### 4.1.1 Included studies

**Figure 3:**
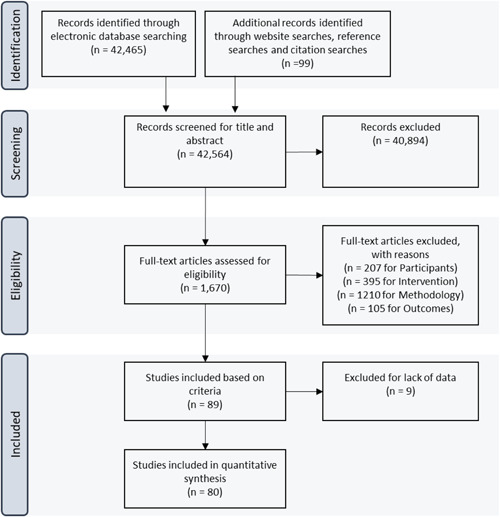
Number of included and excluded papers. Full‐text articles can be excluded for multiple reasons. For instance, the same study can be excluded for not meeting criteria in terms of participants and methodology. N refers to the number of studies

**Figure 4:**
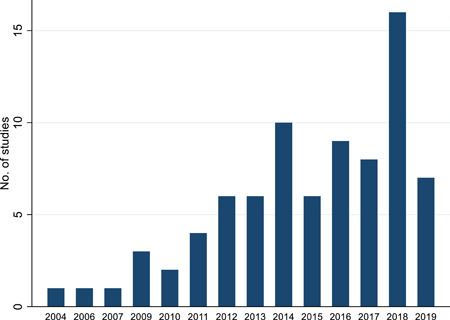
Number of studies in the analysis by year

After applying the search and screening procedures described in the previous sections, 80 studies were selected for the analysis (Figure 3). These studies were conducted in 45 countries, covering all regions of the world including Africa, South Asia, Latin America, Eastern and Western Europe, and East Asia (Table 1) between 2000 and 2019 (Figure 4). While a majority of the studies (79) included were from developing countries, there was one from a high‐income country, as can be seen (Table 2). The data fields extracted from the included studies can be found in Appendix B.

**Table 1:** Number of studies by region

Region	No. of studies
Africa	41
East Asia	7
Europe	3
Latin America	13
South Asia	16
Total	80

**Table 2:** Number of studies by income category

Income category	No. of studies
High income	1
Low income	21
Lower middle income	36
Upper middle income	22
Total	80

**Figure 5:**
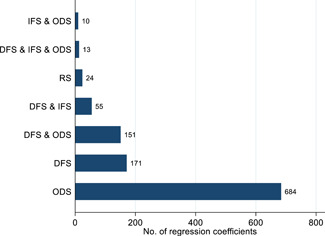
Number of regression coefficients by intervention categories. This figure plots the number of regression coefficients that fit into different intervention categories. Categories are described in detail in Section 3.1. DFS or “Direct Financial Support” includes the direct provision of funding for technological adoption, for example, access to credit and cash transfers. IFS or “Indirect Financial Support” includes support to pay for the cost of the adoption projects without directly providing funding, for example, fiscal incentives and credit assurances. ODS or “Other Direct Support” includes nonpecuniary interventions, for example, technology extension services and provision of management consultancy and training. RS or “Regulations and Standards” includes rules, policies, and characteristics of the environment that affect agents' incentives, for example, the level of competition or the characteristics of contracts

In total, we estimated the standardized effect sizes for 1108 regression coefficients from the selected studies. Figure 5 presents the number of regression coefficients by category of intervention. Given that a study can present multiple results for different interventions, we present the following analysis by regression coefficients and not by study. For instance, if a given study presents a regression coefficient for grant provision on technology adoption and another regression coefficient for training on technology adoption, we present them separately for each intervention. Most regressions (684) assess the effects of “other direct support”—such as provision of training, management consulting services, technology extension services, as well as informational interventions—exclusively. The second‐largest category of interventions is “direct financial support” (171)—which includes grants, loans, insurance, and subsidies, among others—exclusively. There were very few regressions assessing the role of “regulations and standards” or “indirect financial support.”

Some studies assessed the impact of a combination of interventions and were therefore classified in more than one intervention category. For instance, a program analyzing the impact of providing firm owners with both training and contract orders (e.g., Hardy & McCasland, 2018) would fall under “direct financial support” and “other direct support.” Similarly, a program providing loans and training to microfinance clients (e.g., Gine & Mansuri, 2014) would also fall under “direct financial support” and “other direct support.”

The outcome variables presented in the selected studies can be broadly categorized into technology‐adoption outcomes and other firm outcomes. Based on our selection criteria, all included studies reported at least one technology‐adoption outcome. There are 26 studies that have only reported a technology‐adoption outcome and no other firm outcomes. The remaining studies present more than one other firm outcome. Therefore, we have more regression coefficients reported on other firm outcomes than on technology‐adoption outcomes.

**Figure 6:**
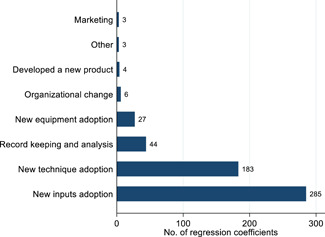
Number of regression coefficients for technology adoption outcomes. This figure plots the number of regression coefficients that are related to the technology adoption outcomes under a broad categorization of technology adoption measures. “Other” includes diversified business activities and spillover adoption

In Figure 6, we present a breakdown of the types of technology‐adoption outcomes used in selected studies. In total, there were 555 regression coefficients measuring the effects of interventions on technology adoption. Since there are too many coefficients to list them individually, we grouped similar ones into broader categories. The attempt is to provide an overview to the reader of the types of variables that have been considered by the studies in our selection. Most of these coefficients analyzed the impact of an intervention on the adoption of a new input or a new technique of production. A small number of regression coefficients analyzed the impact on development of a new product or changes in marketing.

**Figure 7:**
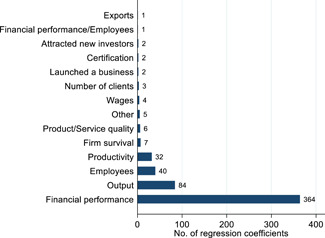
Number of regressions for secondary outcomes. This figure plots the number of regression coefficients that are related to other firm outcomes under a broad categorization of secondary outcomes. Other firm outcomes (or secondary outcomes) are variables that are affected by technology adoption such as financial performance, productivity, and employment, among others. “Other” includes HR index and sought government assistance

Figure 7 presents a breakdown of the other firm outcomes that we consider in our analysis (secondary outcomes). Overall, we find 553 regression coefficients measuring the effects of interventions on other firm outcomes among selected studies. Given the large number of coefficients found, a broad categorization was used to provide an overview to the reader. The categories with the largest number of regression coefficients were financial performance, output, employees, and productivity.

These figures showcase the wide variety of settings, types of interventions, and outcome variables considered in this analysis. The wide range of interventions and outcome variables used does not lend itself to a meta‐regression type of analysis which is often found in other systematic reviews. Therefore, we do not present a meta‐regression as part of this review.

Also for this reason, we were unable to answer the third question stated in the objectives. Given the broad range of interventions and outcomes, an analysis of heterogeneous effects was not possible. In addition, analyzing heterogeneous effects was also hard because only a few studies reported information on variables such as gender and workers' skills.

#### 4.1.2 Excluded studies

There were two rounds of exclusions as described in Figure 3. In the first round, 40,894 studies were excluded based on title and abstract out of 42,564 candidate papers. In the second round, another 1581 studies were excluded based on full text screening. Exclusions in both rounds were based on the four‐element criteria discussed in Section 3.1: intervention, participants, outcomes, and methodology.

For instance, studies were excluded based on the intervention criterion if they did not analyze the effect of credibly exogenous variation in the incentives for firms to adopt a new technology. Thus, we excluded studies that only focused on correlates of technology adoption without an identification strategy to establish causality. Studies were excluded based on participants if they did not analyze effects at the firm level. For example, the study by Hjort and Poulsen (2019) analyzes the impact of adoption at the regional level and was not included in our analysis. Studies were excluded based on outcomes if they did not have a technology adoption outcome variable, for example McKenzie (2017) and Das et al. (2013). Lastly, studies were excluded based on methodology if they did not implement one of the methodologies for causal inference prespecified in our protocol; for example, Conley and Udry (2010) was excluded for this reason.

In addition, of the 89 selected studies that met the criteria, 9 did not report the sample size and/or *SE* and we were unable to receive this information from their authors. These were also excluded for the analysis.

### 4.2 Assessment of reporting biases

We assessed the quality of the evidence in terms of the completeness of reporting in four categories: (1) reporting on key aspects of selection bias and confounding, (2) reporting on spillovers of interventions to comparison groups, (3) reporting on *SE*s, and (4) reporting on Hawthorne effect and collection of retrospective data. The tool followed in the assessments is presented in Appendix C and the results of the assessment in Appendix D.

Most studies report on key aspects of selection bias and confounding, regardless of the methodology used, as presented in Table 3. RCTs typically report on considerations regarding random assignment, baseline characteristics, and attrition. However, there is room for improvement when it comes to reporting on heterogeneous effects. Of the 39 studies reporting heterogeneous effects, 24 do not report evidence that the strategy for choosing groups was planned or announced before the randomization. Among the 30 studies not reporting on the strategy for choosing some groups, only nine report on potential threats to validity of causal claims due to specification searching in the analysis of heterogeneous impacts. Also, there were six studies that report evidence that the strategy for choosing groups was planned or announced before the randomization for some of the groups used to analyze heterogeneous effects but not for others.

**Table 3:** Completeness of reporting assessment (number of studies)

	Completeness of reporting (number of papers)				
		R	NR	NA	Total
	*(1) Reporting on key aspects of selection bias and confounding*				
RCT	(1) The study reports that the assignment was done at random, or describes a random component as part of the assignment procedure.	62	0	0	62
	(2a) If the study shows heterogeneous effects at the group level, it reports some evidence of the strategy for choosing groups before the randomization.	15	24	23	62
	(2b) If the study shows heterogeneous effects at the group level but no evidence of the strategy for choosing groups before the randomization, it reports potential threats to validity of causal claims based on the analysis of heterogeneous impacts.	9	21	32	62
	(3) The study reports baseline characteristics of the treatment and control groups, and/or that overall they are statistically similar.	61	1	0	62
	(4) If there is some difference in baseline characteristics between treated and control groups, the study reports that this difference is accounted for.	35	0	27	62
	(5) If there is attrition, the study reports that the loss of sample units can be considered random and/or intention‐to‐treat estimates.	39	0	0	39
RDD	(1) The study reports that the allocation is based on a threshold in a continuous variable.	0	0	0	0
	(2) The study reports that the participants cannot manipulate the assignment variable.	0	0	0	0
	(3) The study reports that the estimation is robust to different choices of bandwidth.	0	0	0	0
	(4) The study reports that baseline characteristics are overall continuous around the assignment threshold and/or if some baseline characteristic show a discontinuity around the assignment threshold, the study reports that this difference is controlled for when estimating the treatment effects.	0	0	0	0
DiD	(1) The study reports that the outcome variables' trends are parallel before the introduction of the treatment. This could be in the form of a table [some regression‐based test] or graphs.	7	0	0	7
PSM or stat control	(1) The study describes the control method used and it is based on baseline characteristics that are relevant to explain participation.	9	0	0	9
	(2) If the study reports baseline characteristics that are not used for control, it reports that these are overall statistically similar in different groups.	1	2	6	9
IV	1) The paper acknowledges and addresses potential threats to validity (e.g., (violation of exclusion restriction).	2	0	0	2
	(2) The study reports the first‐stage regression F‐statistic.	2	0	0	2
Synthetic control	(1) The study reports that the synthetic control matches the characteristics of the treated units before the treatment.	0	0	0	0
	(2) The study reports robustness to the weights used to measure de difference between the characteristics of the treated unit and the synthetic control.	0	0	0	0
	*(2) Reporting on spillover of intervention to comparison groups*				
	(1) The study reports whether there are likely to be spillovers to comparison group.	49	19	12	80
	(2) If the study reports that there are likely to be spillovers to the comparison group, then it accounts for these in the analysis.	28	4	48	80
	*(3) Reporting of standard errors*				
	(1) If the study report that observations might be independent, this is taken into account by computing the heteroskedasticity‐robust SE. In all other cases, clustered SE have been reported.	68	12	0	80
	*(4) Reporting on Hawthorne effect and collection of retrospective data*				
	(1) If the data are collected in the context of the study, the study reports that the monitoring process did not affect behavior (Hawthorne effect).	13	34	33	80
	(2) If baseline information was collected retrospectively, the study reports this.	13	0	67	80

Abbreviations: NA, not applicable; NR, not reported; R, reported.

Most studies also report on considerations related to spillovers of the interventions to comparison groups and *SE*s. However, of 47 papers collecting data in the context of the study, only 13 discuss whether the monitoring process itself affected behavior (Hawthorne effect).

### 4.3 Synthesis of results

This section presents the standardized effect sizes for all the selected papers. The results are presented using forest plots to allow for visual comparison.

The forest plots show the SMD by study and the associated confidence interval.^2^ The Intervention Category column indicates the type of intervention as described in Section 3.1. DFS or “Direct Financial Support” includes the direct provision of funding for technological adoption, for example access to credit and cash transfers. IFS or “Indirect Financial Support” includes support to pay for the cost of the adoption projects without directly providing funding, for example fiscal incentives and credit assurances. ODS or “Other Direct Support” includes nonpecuniary interventions, for example technology extension services and provision of management consultancy and training. RS or “Regulations and Standards” includes rules, policies, and characteristics of the environment that affect agents' incentives, for example the level of competition or the characteristics of contracts.

To follow the logic presented under Section 1.2 “Potential Channels of Effects” and address our the research questions, we first synthesize our findings on the impact of interventions on technology‐adoption outcomes and then focus on the consequent effects on other firm outcomes.

There is wide variation in the types of interventions examined by included studies. These interventions included training, consulting, information provision, provision of equipment, access to credit, and so forth, to name just a few. There was also a wide range of technology‐adoption outcomes. Due to this situation, we decided it was not proper to conduct analysis of heterogeneous effects and therefore could not achieve the third objective of the review. In addition, the selected papers rarely provided sufficient information for us to address heterogeneity. However, we analyze the effects of interventions separately for agricultural firms and for firms in the manufacturing and services (nonagricultural) sector. This is because the factors impeding technology adoption for agricultural and nonagricultural firms are somewhat different. Information barriers may be more significant for nonagricultural firms relative to agricultural firms. For example, in contrast to agricultural firms, those in the nonagricultural sector often cannot learn about new technologies by observing neighbors. Moreover, nonagricultural firms seldom have any incentive to share information on new technologies with competing firms.

#### 4.3.1 Effects of interventions on technology adoption (research question 1)

##### Manufacturing and services

**Figure 8:**
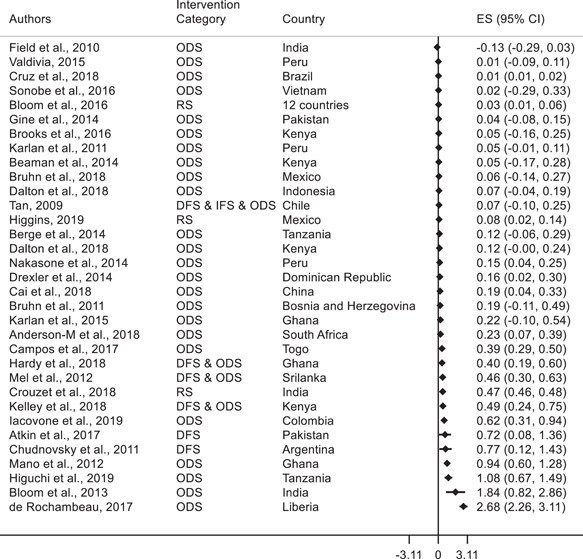
Forest plot of the effects of firm‐level interventions in manufacturing and services on technology adoption. ES is the SMD and 95% CI is the associated confidence interval. DFS, direct financial support; IFS, indirect financial support; ODS, other direct support; RS, regulations and standards; SMD, standardized mean difference.

We analyze 33 studies that focus on technology adoption in manufacturing and services. Figure 8 shows the forest plots along with the standardized effect sizes for the associated studies. Overall, there is mixed evidence on the impact of various interventions at the firm level on technology adoption. Of the 33 studies, 19 find positive and statistically significant standardized effects on technology adoption. The other 14 studies do not find statistically significant effects. We briefly highlight below the interventions that led to statistically significant increases in technology adoption. Note however, that a similar intervention in a different context may not have resulted in statistically significant increases in technology adoption.

There is a wide set of interventions that report a positive effect on technology adoption. These include ICT provision in the form of monitoring and tracking devices in Liberia and Kenya (de Rochambeau, 2017; Kelley et al., 2018), consulting on management and production practices (Bloom et al., 2013; Cruz et al., 2018; Iacovone et al., 2019), and management and business training including accounting, marketing, and finance (Anderson‐Macdonald et al., 2018; de Mel et al., 2012; Drexler et al., 2014; Higuchi et al., 2019; Mano et al., 2012; Nakasone & Torero, 2014). Campos et al. (2017) find that a psychology‐based personal‐initiative‐training approach that teaches a proactive mindset and focuses on entrepreneurial behaviors in Togo had a positive effect on adoption. There is also evidence that increased import competition from China increased measures of technical change across firms in 12 countries (Bloom et al., 2016). However, several other studies that provided business training or management consulting to firms do not find a statistically significant standardized effect (Bruhn & Zia, 2013; Field et al., 2010; Gine & Mansuri, 2014; Karlan & Valdivia, 2011; Valdivia, 2015). Cai and Szeidl (2018) randomized Chinese firms into small groups whose managers held monthly meetings for 1 year and find positive effects of these business networks on firms' management scores.

Direct provision of new techniques of production also led to higher take‐up by firms when they were combined with other interventions. Atkin et al. (2017) provide a new cutting technology to soccer‐ball firms in Pakistan and find low take‐up. However, a subsequent small incentive payment to key employees resulted in positive take‐up of the technology. Hardy and McCasland (2018) randomly seed training in a newly developed weaving technique, and technique‐specific, time‐limited, one‐time contracts among garment making firms in Ghana. They find that random contract offers increase both learning by potential adopters and sharing by incumbent adopters.

Various national policy interventions have also been effective in increasing technology adoption among firms. For example, Chudnovsky et al. (2011) study the Non‐Reimbursable Funds program of the Argentinean Technological Fund (FONTAR) and find positive effects. Crouzet et al. (2019) study the effects of the 2016 Indian demonetization episode, which led to a large but temporary decline in the availability of cash and find a persistent increase in the growth rate of the user base of a new payment technology. Higgins (2019) studies the roll‐out of debit cards in Mexico between 2009 and 2012 and finds that small retailers adopt point‐of‐sale (POS) terminals to accept card payments, which leads other consumers to adopt cards.

##### Agriculture

**Figure 9:**
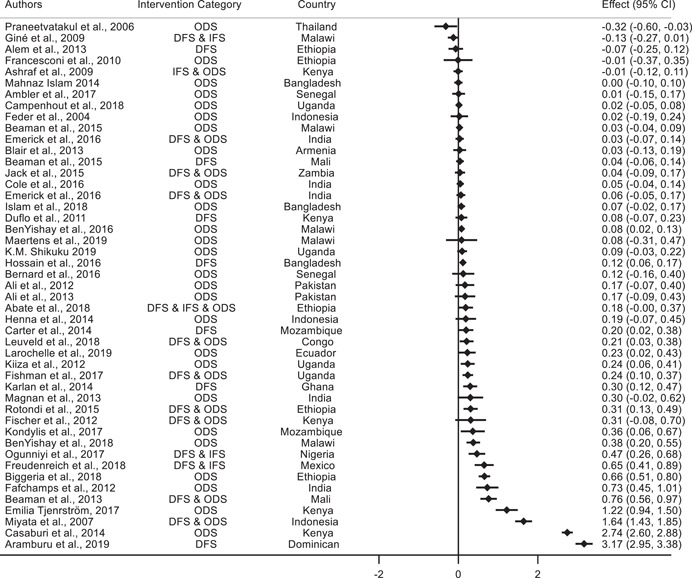
Forest plot of the effects of firm‐level interventions in agriculture on technology adoption. ES is the SMD and 95% CI is the associated confidence interval. DFS, direct financial support; IFS, indirect financial support; ODS, other direct support; RS, regulations and standards; SMD, standardized mean difference

We analyze 47 studies that assess technology adoption as an outcome variable in the agricultural sector. Figure 9 shows the forest plots along with the standardized effect sizes for the associated studies. Similar to the results for manufacturing and services, there is mixed evidence on the impact of various interventions on technology adoption in agriculture. Of the 47 studies, 20 studies find positive and statistically significant standardized effect sizes, and are briefly highlighted below. The other 27 studies do not find statistically significant effects.

For the agricultural sector, several studies focusing on financial support (either direct or indirect) find positive impacts on technology adoption. These interventions include for example, agricultural microcredit in Bangladesh (Hossain et al., 2016), vouchers for fertilizer and improved seeds in Mozambique (Carter et al., 2014), addition of input subsidies to an agricultural extension program in Democratic Republic of Congo (Leuveld et al., 2018), an NGO‐run agricultural input subsidy and extension program in Uganda (Fishman et al., 2017), random provision of fertilizer to rice farmers in Mali (Beaman et al., 2013), nonreimbursable vouchers to partially finance the total cost of improved pastures technology in the Dominican Republic (Aramburu et al., 2019), and access to credit to invest in floating net aquaculture after being relocated due to a reservoir construction project in Indonesia (Miyata & Sawada, 2007).

Studies analyzing the provision of insurance also found positive effects on adoption. For example, Karlan et al. (2014) randomly assign farmers in northern Ghana to receive cash grants, opportunities to purchase rainfall index insurance, or a combination of the two and find that insurance leads to significantly larger agricultural investment and riskier production choices. Freudenreich and Mußhoff (2018) find that bundling hybrid seeds with an insurance scheme increases adoption in Mexico. However, many similar interventions in other contexts do not lead to increased adoption. For example, Giné and Dean (2009) find little evidence that randomized offers of credit and weather insurance in Malawi increased adoption.

Several studies also find positive effects on adoption as a result of learning through agricultural extension services (Rotondi et al., 2015), social networks (BenYishay & Mobarak, 2018; Benyishay et al., 2016; Kondylis et al., 2017; Tjernström, 2017); ICT including sending text messages (Fafchamps & Minten, 2012; Kiiza & Pederson, 2012; Larochelle et al., 2019), and joining value chains (Biggeri et al., 2018). However, there is substantial heterogeneity in learning through social networks. Benyishay et al. (2016) find that other farmers are less willing to learn from female communicators about a new technology. BenYishay and Mobarak (2018) find that the social identity of the communicator influences others' learning and adoption and farmers appear most convinced by communicators who share a group identity with them, or who face comparable agricultural conditions. Tjernström (2017) studies the randomized introduction of a hybrid maize seed in rural Kenyan villages, which induces experimental variation in the information available to farmers through their social networks, and finds that learning from others depends on the soil quality of the farmer.

#### 4.3.2 Effects of interventions on other firm outcomes (research question 2)

Having discussed the effects of various interventions on technology adoption for firms, we next focus on the second research question on the effects of interventions on other firm outcomes: profits, employees, productivity, and yield. In the plots below, we discuss the effects of interventions on other firm outcomes and indicate whether the study also finds a positive effect on technology adoption outcomes.

##### Manufacturing and services

**Figure 10:**
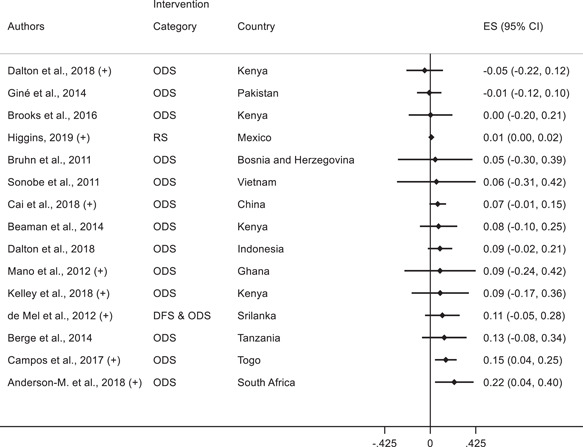
Forest plot of the effects of firm‐level interventions on profits. ES is the SMD and 95% CI is the associated confidence interval. (+) indicates the study reports a positive significant technology adoption outcome. DFS, direct financial support; IFS, indirect financial support; ODS, other direct support; RS, regulations and standards; SMD, standardized mean difference

In manufacturing and services, fifteen studies report profits as an outcome variable and we present the corresponding forest plot in Figure 10. Of these, 3 studies find a positive standardized effect size on profits. Anderson‐Macdonald et al. (2018) measure the impact of marketing and finance skills training for small firms in South Africa; Campos et al. (2017) measure the impact of psychology‐based personal initiative training in Togo; and Higgins (2019) studies a shock to credit card adoption in Mexico and finds an increase in profits for corner stores that adopt POS terminals to accept card payments. It is important to note that these three studies also find a positive effect on technology adoption.

The other 12 studies that report effects on profits find statistically insignificant standardized effect sizes. Some also involved business training (Bruhn & Zia, 2013; Gine & Mansuri, 2014) and five find positive effects of interventions on technology adoption.

**Figure 11:**
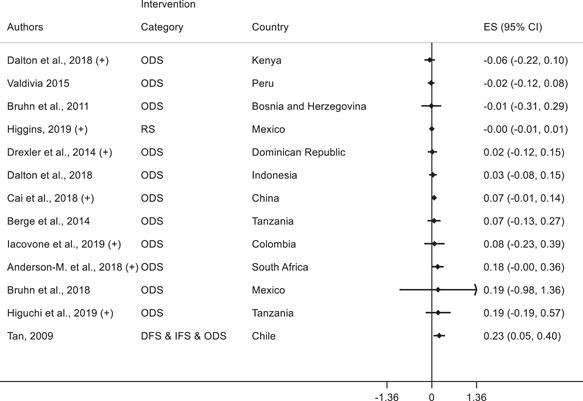
Forest plot of the effects of firm‐level interventions on the number of employees. ES is the SMD and 95% CI is the associated confidence interval. (+) indicates the study reports a positive significant technology adoption outcome. DFS, direct financial support; IFS, indirect financial support; ODS, other direct support; RS, regulations and standards; SMD, standardized mean difference

Regarding employment, 13 studies report on the impact of an intervention on the number of employees as an outcome variable. These are presented in Figure 11. Of the 13 studies, 12 report small and statistically insignificant impacts. Seven of the studies report a positive and statistically significant standardized effect size on technology‐adoption outcomes. The only study that reports a positive impact on the number of employees (although no significant effect on technology adoption) is Tan (2009), which looks at the effects of a SME support program in Chile.

**Figure 12:**
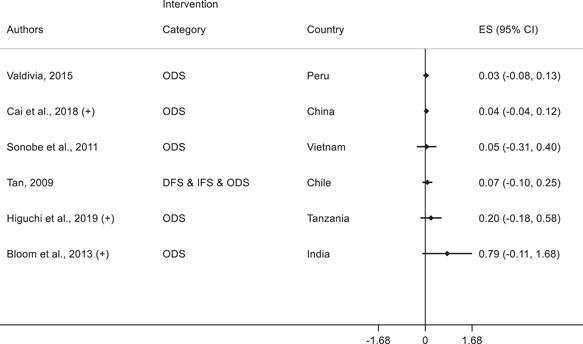
Forest plot of the effects of firm‐level interventions on productivity. ES is the SMD and 95% CI is the associated confidence interval. (+) indicates the study reports a positive significant technology adoption outcome. DFS, direct financial support; IFS, indirect financial support; ODS, other direct support; RS, regulations and standards; SMD, standardized mean difference

In Figure 12, we show the six studies that report results on the effects of interventions on productivity. The studies that have reported on total factor productivity, output per worker, and change in value added were included under this category. None of the papers find a statistically significant standardized effect size on firm productivity at the 95% level, although three report a positive and statistically significant standardized effect size on technology adoption outcomes. Bloom et al. (2013) finds the largest effect size in our selected sample of studies, but the standardized effect size is significant only at the 90% level. Programs that provide training (Valdivia, 2015), learning from peers (Cai & Szeidl, 2018) and other SME support (Tan, 2009) have point estimates (of standardized effect sizes) in the expected direction but are not statistically significant.

##### Agriculture

**Figure 13:**
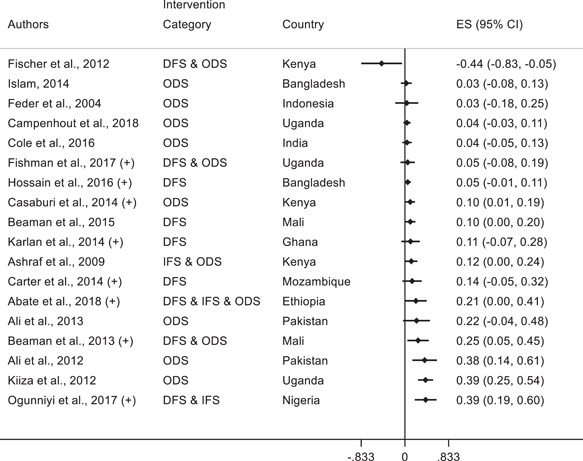
Forest plot of the effects of firm‐level interventions on yields. ES is the SMD and 95% CI is the associated confidence interval. (+) indicates the study reports a positive significant technology adoption outcome. DFS, direct financial support; IFS, indirect financial support; ODS, other direct support; RS, regulations and standards; SMD, standardized mean difference

Out of the sample of selected studies, 18 report effects on yield as on outcome variable. The forest plot is presented in Figure 13. Of the 18 studies, 8 find a statistically significant and positive standardized effect size of interventions on yields and most of these (4 of 8) also find a positive effect on technology adoption outcomes. Another 9 studies find insignificant effects of interventions on yields; 4 of these find positive effects on technology adoption. One study reports a statistically significant and negative standardized effect size on yields (Fischer & Qaim, 2012).

There is mixed evidence that direct financial support to farmers has an effect on yields. On the one hand, direct provision of fertilizers (Beaman et al., 2013), grants to farmers (Beaman et al., 2015), and subsidized agricultural inputs (Ogunniyi et al., 2017) have positive standardized effect sizes on yields. However, other similar interventions do not result in higher yields—for example, an agricultural input subsidy and extension program in Uganda (Fishman et al., 2017), a provision of cash grants and rainfall index insurance in Ghana (Karlan et al., 2014), or an agricultural microcredit in Bangladesh (Hossain et al., 2016).

Other interventions that led to higher yields include farmer field schools in Pakistan that provided skills on integrated pest management (Ali & Muhammad, 2012), the marketing assistance component of Ethiopia's Agricultural Transformation Agency (Abate et al., 2018), NGO‐led services to help farmers adopt and market export crops in Kenya (Ashraf et al., 2009), ICT‐based market information through FM radio stations (Kiiza & Pederson, 2012), and SMS messages with agricultural advice for farmers in Kenya (Casaburi et al., 2019; Van Campenhout et al., 2018).

## 5 DISCUSSION

### 5.1 Overall completeness and applicability of evidence

We reviewed evidence in 80 studies on the impact of interventions on technology adoption and other firm outcomes. All studies include an identification strategy explicitly aiming to estimate causal effects on technology adoption and address threats to internal validity.

Although the studies consider a broad range of interventions, those related to the provision of “Other Direct Support” and “Direct Financial Support” are more common. Relatively few papers studied “Indirect Financial Support” or “Regulation and Standards” interventions.

Regarding outcomes, all selected studies (80) examine the effects on technology‐adoption outcomes as this was embedded in the eligibility criteria. However, only 15 studies estimate effects on profits, 13 on employment, and 6 on productivity.

Due to external‐validity limitations, results in one context cannot necessarily be generalized to another. Indeed, in some cases interventions found to have positive impact in one context did not have a positive impact in other contexts. Adding to the complexity of the results is the wide range in types of interventions. Coming up with a consistent taxonomy of outcome variables is challenging in itself. Given the wide variety of interventions and outcomes, we have opted not to conduct heterogeneity analysis of the results. With all of these caveats in mind, we ask the reader to consider the results presented in the review as suggestive rather than definitive.

### 5.2 Quality of the evidence

Overall, 62 out of the 80 papers selected use a RCT which is the most rigorous methodology to assess threats to validity. The rest use quasi‐experimental methods. Regarding reporting biases, most studies followed best practice when it comes to reporting on selection bias and confounding factors considerations, spillovers of the interventions to the comparison groups, and *SE*s. However, there is room for improvement when it comes to reporting on heterogeneous effects and Hawthorne effects.

### 5.3 Limitations and potential biases in the review process

While we have tried to mitigate many of the potential biases that could affect the results of our study, there were certain aspects of the process that were determined by the constraints of human resources and time. We would like to acknowledge that these could be a potential source of concern.

1. Lack of double coding and double screening for all studies: Resource constraints dictated that we were only able to ensure double screening for the initial batch of 700 papers per person before each screener went on to screen independently. Similarly, given the small number of studies selected, data extraction was completed by one person. This may be a potential source of bias.
2. Limit to studies in English: Due to resource constraints, we confined our electronic and manual search to only the English language. This could potentially be another source of bias.
3. Exclusion of studies: In 46 of the 89 studies, we had to approach the authors either for the missing information or for clarifications. We did not hear back from the author(s) in the case of nine studies.
4. Generalizing across contexts: The purpose of the study was to compile the impact‐evaluation literature on technology adoption in a systematic manner to identify knowledge gaps as well as areas where evidence is strong. One of the challenges of a study of this scope and magnitude lies in the broad variety of contexts, interventions, and outcomes to consider and compile. Given the wide variety, the potential to overlook the many nuances become all the more likely. For this reason, we recommend the reader to take these results as indicative and not conclusive evidence.

### 5.4 Agreements and disagreements with other studies or reviews

Unlike existing systematic reviews about technology adoption that are mostly focused on agricultural firms (Fuglie et al., 2019; Obayelu et al., 2017; Silva et al., 2015; Waddington et al., 2014), this review includes studies from other sectors such as manufacturing (e.g., Atkin et al., 2017; Bloom et al., 2013). It also complements Piza et al. (2016) by including a wider set of interventions but focusing solely on technology adoption. Just as in those studies, we do find interventions that have led to an increase in technology adoption. However, other context‐specific factors seem to matter in how much firms benefit from technology adoption in terms of other firm outcomes. On the whole we find the literature in this area to be still in a nascent stage. There is a need to conduct replications of similar programs across different contexts to have stronger evidence on successful interventions.

## 6 AUTHORS' CONCLUSIONS

### 6.1 Implications for practice and policy

Technology adoption is associated with better economic performance and is considered an important policy goal. Policy‐makers in many countries have incorporated the promotion of firms' competitiveness into their priorities. However, it is unclear what sorts of interventions work in incentivizing firms to adopt new technologies and improve their performance. This review has taken a first step toward filling this knowledge gap by examining the existing evidence on a wide range of interventions that affected firms' incentives for technology adoption in the manufacturing, services, and agriculture sectors. We have also reviewed the evidence on firm performance from these interventions. We have assessed interventions implemented in 45 countries, covering Africa, South Asia, Latin America, Eastern Europe and East Asia. Since the interventions and outcome variables considered in the analysis differed considerably across studies, we have not presented a meta‐regression/meta‐analysis as part of this review. We have, however, presented standardized effect sizes for the various interventions and outcome variables.

We have studied interventions that affect firms' incentives for technology adoption (e.g., adoption of a new input, new technique of production, improved record keeping and analysis, new equipment adoption, and increased knowledge). For the agricultural sector, we have found that studies focusing on financial support (either direct or indirect) – including agricultural microcredit, fertilizer and improved seeds vouchers, input subsidies, provision of fertilizer, vouchers to finance improved technology, and access to credit and insurance – often find positive impacts on technology adoption. However, many similar financial support interventions in other contexts do not lead to increased adoption. In several cases, other direct support, including agricultural extension services, exposure to peer firms through social networks, ICT, and value chains led to increased technology adoption.

In manufacturing and services, studies have found that a wide set of interventions led to positive effects on technology adoption depending on the setting. In several cases, interventions such as direct provision of new techniques, consulting on management and production practices, management and business training including accounting, marketing, finance, and psychology‐based personal initiative training increased technology adoption. However, in other contexts, studies that provided business training or management consulting to firms did not find a statistically significant standardized effect on technology adoption.

We have also studied the effects of interventions in our sample of papers on other firm performance measures. We find that studies that report a positive effect on technology‐adoption outcomes do not necessarily find a corresponding positive effect on other firm outcomes. Overall, in the agricultural sector, there is mixed evidence that direct financial support to farmers has an effect on yields. Other interventions that have led to higher yields include farmer field schools, marketing assistance, NGO‐led services to help farmers adopt and market export crops, ICT‐based market information and SMS messages with agricultural advice for farmers. For firms in manufacturing and services, most of the studies we reviewed find statistically insignificant effects on profits, productivity, and employment.

Taken together, our results suggest that while various interventions can generate positive impacts on technology adoption among firms, these effects tend to be context‐specific. We have found that the effects on farm yields, firm profits, productivity, and employment also tend to be mixed. Therefore, overall, we are unable to say that one group of interventions led to a higher impact and can be favored over others. Policy‐makers must be careful in interpreting these results, as the same intervention cannot be assumed to work equally well across contexts and needs to be adjusted to each specific regional context. That said, policymakers need to try out different types of interventions to see what works in their setting. We also emphasize that a statistically insignificant finding for a type of intervention in a particular context does not mean that all interventions of that type are unworthy of consideration. Rather, attention should be paid to how programs can be improved and better tailored to particular environments to achieve better outcomes.

### 6.2 Implications for research

The results of this review strongly point to the need for additional research to understand: (i) what sorts of interventions work in inducing firms to adopt new technologies, and (ii) the effects of technology adoption on other measures of firm performance. We have found that the effects of interventions can vary widely across contexts. There is a need to conduct more replications of similar programs across different contexts and to examine closely the processes of implementation in different studies. This will help policy‐makers understand which interventions work and why.

The three primary recommendations for future research based on this review are:

1. Identify intervention areas that are less studied. While some interventions classified as “Other Direct Support” and “Direct Financial Support” such as consulting, training, grants, and subsidies have received significant attention from researchers, the impact of “Indirect Financial Support” including interventions such as access to credit, and incentive payments, as well as “Regulations and Standards” have been less studied in the context of technology adoption.
2. It would be helpful for researchers to provide all the necessary information required to compute SMDs. We had to contact 46 of the 89 selected studies for either clarifications or information to compute SMDs. Reporting on the results with all the appropriate information can go a long way in reducing the effort required to collate the information in a meaningful way.
3. Based on the assessment of reporting biases, researchers could improve reporting on heterogeneous effects by including some evidence of the strategy for choosing groups before the randomization or potential threats to validity of causal claims based on the analysis of heterogeneous impacts. Researchers can also improve reporting on Hawthorne effects by reporting whether the monitoring process may affect behavior.

Although this review only focused on interventions that induce firms to adopt new technology, researchers also need to focus on the reasons behind low technology adoption by firms in developing countries. As a broad categorization, reasons for low adoption may be (i) internal to the firm, (ii) on the input side, and (iii) on the output side (Verhoogen, 2020). Considering factors internal to the firm, there may be low adoption of new technologies because firms may not be profit maximizing, may lack information, or may not have the capability to apply the information in practice. There is a need for more research analyzing interventions that alleviate these concerns such as the effects of increasing competition, or the effects of learning through various channels, for instance through interactions with peers, customers, suppliers, employees, or consultants. On the input side, newer technology often requires highly skilled workers and high‐quality inputs, which may be scarce and expensive. Similarly, on the output side, demand conditions may affect the incentives of firms to adopt (or not) new technologies. Often, customers in richer countries have preferences for higher‐quality goods, which may require the application of new technologies. In this respect, there is a need for increased research on the effects of exporting or participation in global value chains on technology adoption. Understanding barriers to technology adoption will also help researchers design interventions that help alleviate them, thereby leading to increased adoption.

Overall, we believe that fruitful areas of future research would include both an understanding of barriers to technology adoption and interventions that lead to increased adoption through removal of those barriers.

## INFORMATION ABOUT THIS REVIEW

### Review authors

**Lead review author:**

The lead author is the person who develops and co‐ordinates the review team, discusses and assigns roles for individual members of the review team, liaises with the editorial base and takes responsibility for the on‐going updates of the review

**Table:** 

**Name:**	Ana Goicoechea
Title:	Senior Economist
Affiliation:	World Bank Group
Address:	1818 H Street NW
City, State, Province or County:	Washington DC
Postal Code:	20433
Country:	USA
Phone:	+1‐202‐458‐9781
Email:	agoicoechea@worldbank.org

**Co‐authors (in alphabetical order):**

**Table:** 

**Name:**	David Alfaro‐Serrano
Title:	PhD Candidate
Affiliation:	Columbia University
Address:	420 W. 118th St., MC 3308
City, State, Province or County:	New York, NY
Postal Code:	10027
Country:	USA
Email:	da2628@columbia.edu

**Table:** 

**Name:**	Tanay Balantrapu
Title:	Research Analyst
Affiliation:	World Bank Group
Address:	1818 H Street NW
City, State, Province or County:	Washington DC
Postal Code:	20433
Country:	USA
Email:	tbalantrapu@ifc.org

**Table:** 

**Name:**	Ritam Chaurey
Title:	Assistant Professor
Affiliation:	Johns Hopkins University, School of Advanced International Affairs (SAIS)
Address:	1717, Massachusetts Avenue NW
City, State, Province or County:	Washington D.C.
Postal Code:	20036
Country:	USA
Email:	rchaurey@jhu.edu

**Table:** 

**Name:**	Eric Verhoogen
Title:	Professor
Affiliation:	Economics and SIPA, Columbia University
Address:	420 W. 118th St., Room 1022
City, State, Province or County:	New York, NY
Postal Code:	10027
Country:	USA
Email:	eric.verhoogen@columbia.edu

**Research Assistants (in alphabetical order):**

**Table:** 

**Name:**	Snigdha Dewal
Title:	Research Assistant
Affiliation:	
Address:	
City, State, Province or County:	Washington DC
Postal Code:	2009
Country:	USA

**Table:** 

**Name:**	Wentian Jiang
Title:	Research Assistant
Affiliation:	World Bank Group
Address:	1818 H Street NW
City, State, Province or County:	Washington DC
Postal Code:	20433
Country:	USA

**Table:** 

**Name:**	Tommy Jungyul Kim
Title:	Research Analyst
Affiliation:	World Bank Group
Address:	1818 H Street NW
City, State, Province or County:	Washington DC
Postal Code:	20433
Country:	USA

**Table:** 

**Name:**	Jincy Wilson
Title:	Research Assistant
Affiliation:	World Bank Group
Address:	1818 H Street NW
City, State, Province or County:	Washington DC
Postal Code:	20433
Country:	USA

### Roles and responsibilities

All authors contributed to the writing and revising of this review. Ritam Chaurey, Ana Goicoechea, and Eric Verhoogen provided content and methodological expertise. Tanay Balantrapu led the information retrieval and statistical analysis. David Alfaro‐Serrano led the development of the search strategy and protocol. Advice on information retrieval was kindly offered by John Eyers.

In particular, roles and responsibilities were distributed as follows (names listed in alphabetical order):

- *Content*: All coauthors.
- *Systematic review methods*: All coauthors.
- *Statistical analysis*: Tanay Balantrapu and Ritam Chaurey.
- *Information retrieval*: Tanay Balantrapu, Snigdha Dewal, Wentian Jiang, Tommy Kim, and Jincy Wilson.

### SOURCES OF SUPPORT

We thank Hugh Waddington of 3ie, and Caio Piza of the World Bank Group, for guidance and support. The study has been funded by Competitiveness Policy Evaluation Lab of the World Bank Group with contributions from the facility for Investment Climate Advisory Services (FIAS) and the United States Agency for International development (USAID).

### DECLARATIONS OF INTEREST

There are no conflicts of interest identified at the time of the review.

### PLANS FOR UPDATING THE REVIEW

Any of the coauthors may consider updating the review in 2 years' time or when there has been considerable advances in the literature.

### AUTHOR DECLARATION

#### Authors' responsibilities

By completing this form, you accept responsibility for maintaining the review in light of new evidence, comments and criticisms, and other developments, and updating the review at least once every 5 years, or, if requested, transferring responsibility for maintaining the review to others as agreed with the Coordinating Group. If an update is not submitted according to agreed plans, or if we are unable to contact you for an extended period, the relevant Coordinating Group has the right to propose the update to alternative authors.

#### Publication in the Campbell Library

The Campbell Collaboration places no restrictions on publication of the findings of a Campbell systematic review in a more abbreviated form as a journal article either before or after the publication of the monograph version in *Campbell Systematic Reviews*. Some journals, however, have restrictions that preclude publication of findings that have been, or will be, reported elsewhere, and authors considering publication in such a journal should be aware of possible conflict with publication of the monograph version in *Campbell Systematic Reviews*. Publication in a journal after publication or in press status in *Campbell Systematic Reviews* should acknowledge the Campbell version and include a citation to it. Note that systematic reviews published in *Campbell Systematic Reviews* and co‐registered with the Cochrane Collaboration may have additional requirements or restrictions for co‐publication. Review authors accept responsibility for meeting any co‐publication requirements.

## Figures and Tables

**Figure A1:**
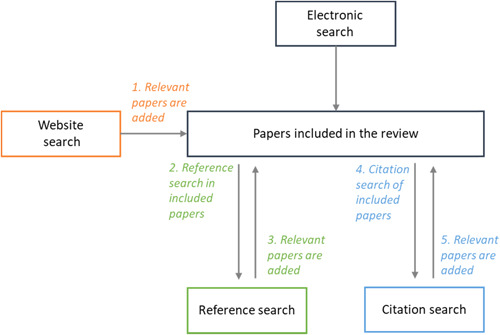
Manual search procedures

## Footnotes

1 We use the World Bank's World Development Indicators classification of countries into low, lower middle, upper middle and high. We collectively refer to all countries not in the high‐income group as “developing.”

2 The “metan” command on Stata is used to generate the forest plots. The average pooled effect is directly computed by the command. The average pooled effect here refers to a within‐study mean.

## APPENDIX A MANUAL SEARCHES

In addition to the electronic search, we conducted a manual search implemented between May, 2018 and June, 2020. It entailed the following three procedures:

1. Search of websites of organizations working on the topic
2. Review of the references of each of the papers included after full text screening
3. Review of citations of the included papers

The three procedures were executed as presented in Figure A1. Once step 5 was completed, steps 2‐5 were repeated to conduct reference and citation searches on additional relevant papers until no new relevant papers were found. For each procedure, the screening procedure outlined in Figure 2 of the protocol was used.

1. *Website Search*
  The website search involved screening 2258 papers, and 32 of them were included based on full text screening. Ten organizations were selected when the protocol was originally conceived. The search process of the websites imitated the database search procedure as much as possible, but the search strategy had to be tailored to each website. For example, some websites only allowed a keyword search while some allowed for an advanced search. The search method of each website is detailed in Table A1 below.

2. *Reference search*
  For each of the included papers, the references cited were first screened by title and abstract and then by full text, using the criteria described in section 3.1. Abstracts and full texts were accessed through google scholar and the World Bank/IMF electronic library.
3. *Citation search*
  Google Scholar was the primary tool used for the citation search. For each paper, a search within the citation results was conducted using the keywords technology adoption. Given that complex Boolean searches do not function on Google Scholar search, and that the search could not be specified to either the title or abstract, technology adoption was used as the search term. This search term prioritized papers using the phrase “technology adoption,” but also yielded results that use the term “technology” or “adoption” prominently. The citation search relied on the Google Scholar algorithm to prioritize relevant results, and the search would be cut off once relevant papers stopped appearing. For example, suppose that a certain paper had 1000 citations and searching “technology adoption” within the citations yielded 300 results. After a few pages of results, the results begin to be less and less relevant, and the screening would stop once the screener judged that no subsequent results were likely to be included.

**Table A1:** Search methods by website

#	Website	Search Boolean	Approach	Results
1	OECD	from (Abstract contains ‘technolog* OR manag* OR innovat* OR practice*') from (contains ‘en') AND from (Abstract contains ‘(adopt* OR diffus* OR chang* OR alter*)') AND from (IGO collection contains ‘OECD') with type(s) subtype/journal OR subtype/article OR subtype/workingpaperseries OR subtype/workingpaper published between 2003 and 2018	abstract search using advanced search in OECD iLibrary	574
2	3ie	(technolog* OR manag* OR innovat* OR practice*) AND (adopt* OR diffus* OR chang* OR alter*)	keyword search in the Impact Evaluation Repository	193
3	AfDB ‐ website search	(technolog* OR manag* OR innovat* OR practice*) AND (adopt* OR diffus* OR chang* OR alter*)	keyword search in its website search bar	43
4	AfDB ‐ publications search	technology	keyword search in working paper series, under publications	278
5	ATAI	none	No search, manual screening of titles under research publications	30
6	AEA RCT registry	four separate searches: technolog*, manag*, innov*, and practice*	four separate searches were made under advanced search – abstract	639
7	ADB	(technolog* OR manag* OR innovat* OR practice*) AND (adopt* OR diffus* OR chang* OR alter*)	keyword search, after selecting “Papers and Briefs”	96
8	IADB	four separate searches: technolog*, manag*, innov*, and practice*	four separate searches under working papers and co‐publications	337
9	DFID	(technolog* OR manag* OR innovat* OR practice*) AND (adopt* OR diffus* OR chang* OR alter*)	keyword search under publications	20
10	USAID	(technolog* OR manag* OR innovat* OR practice*) AND (adopt* OR diffus* OR chang* OR alter*)	in “Development Experience Clearinghouse” search in title + language English + document type (journal article, conference proceedings/papers)	48

## APPENDIX B ARTICLE CODING AND DATA EXTRACTION

**Table:** Table B1: Fields included in article coding and data extraction

Publication details	Author(s) Year of publication Full citation Publication type: journal article, working paper, book Author(s) affiliation(s) Funding sources for the research
Context of intervention or program	Region: EAP, ECA, LAC, MNA, SAR, SSA Country Declared goal of the intervention If it is a government deliberate intervention, ministry in charge If it is a deliberate intervention, agency in charge of implementation Where did the intervention take place? Period considered in the study Location: urban, rural Funding source(s) for the intervention Total cost of the intervention (US dollars)
Population in the study	If the intervention targets a specific type of unit, definition of target Unit of analysis in the study Average annual output of the productive units Average annual sales of the productive units Average employment of the productive units Sector(s) of operation Fraction of the productive units owned, led, or operated by female individuals. Average fraction of high‐skill workers in units of production Average fraction of female workers in units of production Ownership composition of productive units
Intervention details	Name of the intervention (if any) and brief description Does it include an indirect financial support component? Does it include a direct financial support component? Does it include other direct support component? Does it include a component affecting regulation and standards?
Study design	Methodology: Description of sample selection procedure, including units and sample size for treatment, exposed, and comparison groups If reported separately, treatment group sample size If reported separately, comparison group sample size If reported separately, indirectly exposed group sample size Total sample size If reported separately, attrition in treatment group If reported separately, attrition in comparison group If reported separately, attrition in indirectly treated group Overall attrition Frequency of outcome data collection Total number of data collection periods including baseline Baseline period Intervention periods Follow up periods Duration of intervention Spillovers Contamination
Data for analysis	Treatment variable (indicate units or dichotomous variable values) Outcome variable (If reported for different groups, for example, average wages for male and female employees‐ consider them as different outcomes. Indicate units or dichotomous variable values) Technology adoption outcome? (yes/no) Number of observations N (total sample size) Nc (comparison group sample size) Nt (treatment group sample size) MeanC_Pre: Preintervention comparison group mean MeanC_Post: Post‐intervention comparison group mean sdC_Pre: Preintervention comparison group standard deviation sdC_Post: Post‐intervention comparison group standard deviation MeanT_Pre: Preintervention treatment group mean MeanT_Post: Post‐intervention treatment group mean sdT_Pre: Preintervention treatment group standard deviation sdT_Post: Post‐intervention treatment group standard deviation sdpooled_pre: pooled standard deviation between the treatment and control groups with equal weights preintervention Treatment effect estimated: ITT, ATET, ATE, LATE Model (fixed effects, random effects, etc.) Effect estimate (difference in means) Effect estimate standard error Effect estimate t‐statistic SMD (standardized mean difference across treatment and control groups computed using the treatment effect size and standard deviation) SE(SMD) (Standard error of the standardized mean difference) R‐square F test RR (relative risk ratio) SE(RR) (standard error of the relative risk ratio) P value (p value of the treatment effect) Likelihood ratio Log likelihood ratio Chi square I square Random effects If dichotomous outcome: number of “successes” in control group If dichotomous outcome: number of “successes” in treatment group If dichotomous outcome: number of “failures” in control group If dichotomous outcome: number of “failures” in treatment group Effect size adjustment: adjusted or unadjusted (i.e., using multivariate analysis) treatment effect. Where adjusted analysis, list additional covariates included in model.

## APPENDIX C TOOL FOR ASSESSMENT OF REPORTING BIASES

We assessed reporting biases with a modified version of the 3ie risk of bias tool (Hombrados & Waddington, 2012). The tool covers four dimensions: (1) Reporting on key aspects of selection bias and confounding, (2) Reporting on spillovers of intervention to comparison groups, (3) Reporting on *SE*s, and (4) Reporting on Hawthorne effects and baseline data. For each of these dimensions, a set of considerations were defined and presented in Table C1. The question whether the study addresses each consideration can receive one of three answers: “reported,” “not reported,” or “not applicable.”

**Table C1:** Tool for assessment of reporting biases

	*(1) Reporting on key aspects of selection bias and confounding*
RCT	(1) The study reports that the assignment was done at random or describes a random component as part of the assignment procedure.
	(2a) If the study shows heterogeneous effects at the group level, it reports some evidence of the strategy for choosing groups before the randomization.
	(2b) If the study shows heterogeneous effects at the group level but no evidence of the strategy for choosing groups before the randomization, it reports potential threats to validity of causal claims based on the analysis of heterogeneous impacts.
	(3) The study reports baseline characteristics of the treatment and control groups, and/or that overall they are statistically similar
	(4) If there is some difference in baseline characteristics between treated and control groups, the study reports that this difference is accounted for.
	(5) If there is attrition, the study reports that the loss of sample units can be considered random and/or intention‐to‐treat estimates.
RDD	(1) The study reports that the allocation is based on a threshold in a continuous variable.
	(2) The study reports that the participants cannot manipulate the assignment variable.
	(3) The study reports that the estimation is robust to different choices of bandwidth.
	(4) The study reports that baseline characteristics are overall continuous around the assignment threshold and/or if some baseline characteristic show a discontinuity around the assignment threshold, the study reports that this difference is controlled for when estimating the treatment effects.
DiD	(1) The study reports that the outcome variables' trends are parallel before the introduction of the treatment. This could be in the form of a table [some regression‐based test] or graphs.
PSM or stat control	(1) The study describes the control method used and it is based on baseline characteristics that are relevant to explain participation.
	(2) If the study reports baseline characteristics that are not used for control, it reports that these are overall statistically similar in different groups.
IV	(1) The paper acknowledges and addresses potential threats to validity (e.g., violation of exclusion restriction).
	(2) The study reports the first‐stage regression F‐statistic.
Synthetic control	(1) The study reports that the synthetic control matches the characteristics of the treated units before the treatment.
	(2) The study reports robustness to the weights used to measure de difference between the characteristics of the treated unit and the synthetic control.
	*(2) Reporting on spillover of intervention to comparison groups*
	(1) The study reports whether there are likely to be spillovers to comparison group.
	(2) If the study reports that there are likely to be spillovers to the comparison group, then it accounts for these in the analysis.
	*(3) Reporting of standard errors (SE)*
	(1) If the study report that observations might be independent, this is taken into account by computing the heteroskedasticity‐robust SE. In all other cases, clustered SE have been reported.
	*(4) Reporting on Hawthorne effects and collection of retrospective data*
	(1) If the data are collected in the context of the study, the study reports that the monitoring process did not affect behavior (Hawthorne effects).
	(2) If baseline information was collected retrospectively, the study reports this.

Abbreviations: DiD, difference in differences; IV, instrumental variables; PSM, propensity score matching; RCT, randomized control trial; RDD, regression discontinuity design.

## APPENDIX D RESULTS OF ASSESSMENT OF REPORTING BIASES

**Table:** Table D1: Assessment of reporting biases

	(1) Reporting on key aspects of selection bias and confounding											(2) Spillovers		(3) *SE*	(4) Hawthorne and data	
Short citation	RCT item 1	RCT item 2a	RCT item 2b	RCT item 3	RCT item 4	RCT item 5	DiD item 1	PSM item 1	PSM item 2	IV item 1	IV item 2	Item 1	Item 2	Item 1	Item 1	Item 2
Abate et al. (2018)	R	R	R	R	R	NA						R	R	R	NR	NA
Alem and Broussard (2013)							R	R	NA			NA	NA	R	NA	NA
Ali and Muhammad (2012)								R	NA			NR	NR	NR	NA	NA
Ali and Rahut (2013)								R	NR			NA	NA	NR	NA	NA
Ambler et al. (2017)	R	NA	NA	R	R	R						R	R	R	NA	NA
Aramburu et al. (2019)	R	R	R	R	NA	NR						R	R	R	NR	NA
Ashraf et al. (2009)	R	NR	NR	R	R	R						R	NA	R	NR	NA
Atkin et al. (2017)	R	NA	NA	R	R	R						R	R	R	NA	NA
Beaman et al. (2013)	R	NA	NA	R	R	R						NR	NR	R	NR	NA
Beaman et al. (2014)	R	NA	NA	R	R	R						R	R	R	NA	NA
Beaman et al. (2015) (Malawi)	R	NR	NR	R	R	NA						R	R	R	NR	NA
Beaman et al. (2015) (Mali)	R	R	NA	R	NA	R						R	R	R	R	NA
Benyishay et al. (2016)	R	NA	NA	R	R	R						R	NA	R	R	NA
BenYishay and Mobarak (2018)	R	NR	NR	R	R	R						NA	NA	R	R	NA
Berge et al. (2014)	R	NR	NR	R	NA	R						R	NA	R	NR	NA
Bernard et al. (2016)	R	NA	NA	R	NA	NA						R	R	R	NA	NA
Biggeri et al. (2018)								R	NA			R	NA	NR	NA	R
Blair et al. (2013)	R	NA	NA	R	NA	NA						R	NA	R	NR	R
Bloom et al. (2013)	R	NA	NA	R	R	R						R	R	R	R	NA
Bloom et al. (2016)										R	R	R	R	R	NA	NA
Brooks et al. (2016)	R	NA	NA	R	R	R						R	NA	R	NA	NA
Bruhn and Zia (2013)	R	R	R	R	NA	R						NR	NA	R	NR	NA
Bruhn et al. (2018)	R	R	NA	R	R	R						NR	NA	R	NR	NA
Cai and Szeild (2018)	R	R	R	R	NA	R						NA	NA	R	R	NA
Van Campenhout et al. (2018)	R	NR	NR	R	NA	R						R	NR	NR	NR	NA
Campos et al. (2017)	R	R	R	R	NA	R						NR	NA	R	R	NA
Carter et al. (2014)	R	NA	NA	R	R	R						R	R	R	NA	R
Casaburi et al. (2019)	R	R	NA	R	R	R						R	R	R	NA	NA
Chudnovsky et al. (2011)							R					NR	NA	R	NA	R
Cole and Fernando (2016)	R	NR	NR	R	NA	R						R	R	R	NR	NA
Crouzet et al., 2018							R					NA	NA	R	NA	NA
Cruz et al. (2018)										R	R	NA	NA	R	NA	NA
Dalton, Zia, et al. (2018) (Indonesia)	R	R	R	R	NA	R						R	NA	R	NR	NA
Dalton, Pamuk, et al. (2018) (Kenya)	R	NA	NA	R	R	R						NR	NA	R	NR	NA
de Rochambeau (2017)	R	NR	NR	R	NA	NA						NR	NA	R	R	NA
Drexler et al. (2014)	R	R	NA	R	R	R						R	R	R	R	NA
Duflo et al. (2011)	R	NA	NA	R	R	R						R	NA	R	R	NA
Emerick et al. (2016a) (AER)	R	NA	NA	R	NA	R						R	R	R	NA	R
Emerick et al. (2016b) (WB)	R	NA	NA	R	R	NA						NR	NA	R	NR	NA
Emilia Tjenrström (2017)	R	NR	NR	R	R	NA						R	R	R	NR	R
Fafchamps and Minten (2012)	R	NR	NR	R	NA	R						R	R	R	NA	NA
Feder et al. (2003)							R					NA	NA	R	NA	NA
Field et al. (2010)	R	NR	NR	R	NA	R						NR	NR	R	NR	NA
Fischer and Qaim (2012)								R	R			R	R	R	NA	NA
Fishman et al. (2017)	R	NR	NR	R	NA	R						R	NA	R	NA	NA
Francesconi and Heerink (2010)								R	NR			R	NA	NR	NA	NA
Freudenreich and Mußhoff (2018)	R	NR	NR	R	R	NA						NR	NA	NR	NR	R
Giné and Dean (2009)	R	NR	NR	R	R	NA						R	NA	R	NR	NA
Gine and Mansuri (2014)	R	R	NA	R	NA	R						NR	NA	R	NR	NA
Hardy and McCasland (2018)	R	NA	NA	R	R	NA						R	R	R	NR	NA
Henna et al. (2014)	R	NR	R	R	R	NA						NA	NA	R	R	NA
Higuchi et al. (2019)	R	NA	NA	R	R	R						R	R	R	NR	NA
Tan (2009)							R					R	NA	NR	NA	NA
Hossain et al. (2016)	R	NR	NR	R	R	R						R	NA	R	NA	NA
Iacovone et al. (2019)	R	NA	NA	R	R	R						R	NA	R	NR	NA
Islam et al. (2018)	R	NR	NR	R	NA	NA						R	R	R	NA	NA
Jack et al. (2015)	R	NA	NA	R	NA	R						R	NA	R	NA	NA
Shikuku (2019)								R	NA			NA	NA	R	NR	NA
Karlan and Valdivia (2011)	R	NR	NR	R	NA	R						NR	NA	R	NR	NA
Karlan et al. (2014)	R	NR	NR	R	R	NR						NR	NA	R	R	R
Karlan et al. (2015)	R	R	NA	R	NA	R						R	NA	R	R	NA
Kelley et al. (2018)	R	NA	NA	R	NA	NA						NR	NA	R	NR	NA
Kiiza and Pederson (2012)								R	NA			NA	NA	R	NA	NA
Kondylis et al. (2017)	R	NR	R	R	R	R						R	R	R	NR	R
Larochelle et al. (2019)	R	NA	NA	R	R	R						R	NA	R	NA	NA
Leuveld et al. (2018)	R	NR	R	R	R	R						R	R	R	NA	NA
Macdonald et al. (2018)	R	R	NA	R	R	R						R	NA	R	NR	NA
Maertens et al. (2018)	R	NR	NR	NR	NA	NR						NR	NA	R	NR	NA
Magnan et al. (2013)	R	NR	NR	R	R	NA						R	R	NR	NR	NA
Mahnaz Islam (2014)	R	NR	NR	R	R	R						R	R	R	NR	R
Mano et al. (2012)	R	NR	NR	R	NA	R						R	R	R	R	NA
Valdivia (2015)	R	R	NA	R	NA	R						NR	NA	R	NR	NA
de Mel et al. (2012)	R	NA	NA	R	NA	R						R	NA	R	NR	NA
Miyata and Sawada (2007)												NA	NA	NR	NA	R
Nakasone and Torero (2014)	R	NA	NA	R	R	NR						NR	NA	R	NR	R
Ogunniyi et al. (2017)								R	NA			NA	NA	NR	NA	NA
Praneetvatakul and Waibel (2006)							R					R	NA	NR	NA	NA
Rotondi et al. (2015)	R	R	NA	R	R	R						R	R	R	NA	R
Higgins (2019)							R					R	R	R	NA	NA
Sonobe et al. (2011)	R	NA	NA	R	NA	NA						NR	NA	NR	NR	NA

Abbreviations: NA, not applicable; NR, not reported; R, reported.
